# First report of canine parvovirus molecular detection in Bangladesh

**DOI:** 10.14202/vetworld.2021.1038-1043

**Published:** 2021-04-29

**Authors:** F. M. Yasir Hasib, Sharmin Akter, Sharmin Chowdhury

**Affiliations:** 1 Department of Pathology and Parasitology, Faculty of Veterinary Medicine, Chattogram Veterinary and Animal Sciences University, Khulshi, Chattogram 4225, Bangladesh; 2 Department of Medicine and Surgery, Faculty of Veterinary Medicine, Chattogram Veterinary and Animal Sciences University, Khulshi, Chattogram 4225, Bangladesh

**Keywords:** Bangladesh, canine parvovirus, CPV2a, CPV2b, CPV2c

## Abstract

**Background and Aim:**

Canine parvovirus (CPV) is the most important cause of mortality in dogs in many parts of the world. Clinical cases exhibit characteristic signs, including foul-smelling bloody diarrhea, vomiting, fever, and dehydration. This study assessed field and vaccine variants of parvovirus in the Chattogram metropolitan area, Bangladesh. The investigation also aimed to identify risk factors for this disease. This research is the first to identify the presence of CPV in Bangladesh through molecular examination.

**Materials and Methods:**

From October to December 2019, a total of 100 dogs were included in the study. Rectal swabs were taken from all dogs. Twenty dogs showed clinical signs of parvovirus. All clinically affected animals along with 20 randomly selected healthy dogs were tested using amplification refractory mutation system (ARMS)-polymerase chain reaction (PCR) to identify variants from the samples. Logistic regression model analysis was performed to determine the possible risk factors for CPV.

**Results:**

ARMS-PCR showed the presence of all three variants, CPV2a, CPV2b, and CPV2c, in clinically ill dogs, and vaccines available in the study area showed either CPV2a or CPV2b strain. The CPV2c variants showed a higher incidence than the other variants. All apparently healthy animals tested were molecularly negative. Multivariable logistic regression model (generalized linear mixed model) indicated that exotic breeds were 3.83 times more likely to be infected by CPV than local breeds. Furthermore, dogs reared in semi-intensive and extensive management systems were 3.64 and 3.79 times more likely to be infected, respectively, than those reared in an intensive management system.

**Conclusion:**

These findings provide practitioners and pet owners information on the occurrence of different variants and help design effective prevention strategies for CPV infection.

## Introduction

Canine parvovirus (CPV) is a single-stranded DNA virus that is highly contagious among domestic dogs and other carnivores [1,2]. The virus is highly resistant to heat and common disinfectants. It can remain viable for up to 3 months at room temperature (25°C) [3]. CPV is a member of the family Parvoviridae and is a major cause of viral-induced mortality in dogs [4]. CPV shows several characteristic clinical signs, including foul-smelling bloody diarrhea, fever, vomiting, lethargy, anorexia, and for puppies, heart failure leading to death [5]. After initial identification in the 1970s, three antigenic variants (CPV2a, CPV2b, and CPV2c) have been discovered to date [6]. These variants emerged through mutation in the VP2 coding gene [7]. CPV is distributed worldwide and vaccination is a widely accepted method for prevention of this disease [8]. Diagnosis of the disease is possible using several methods, including serological methods, such as enzyme-linked immunosorbent assay and hemagglutination inhibition test, and molecular techniques, such as polymerase chain reaction (PCR), real-time PCR (RT-PCR), minor binding groove assays, single-nucleotide polymorphism, restriction fragment length polymorphism, and amplification refractory mutation system PCR (ARMS-PCR) [5,9-12]. ARMS-PCR is an easy and efficient PCR technique for the diagnosis of CPV infection, which differentiates variants without sequencing [13]. Modified live vaccines containing the CPV2a and CPV2b variants are available worldwide for disease prevention. The CPV2b variants are used more frequently than the CPV2a variants for developing vaccines [14-16].

In Bangladesh, no molecular confirmation of the presence of CPV is available, although diagnosis with a rapid detection kit suggests endemic occurrence [17-20]. Pet practitioners regularly find CPV cases almost year-round and use a standard vaccination strategy for prevention.

This study was conducted to compare circulating field and vaccine variants in the study area using molecular techniques for the 1^st^ time in Bangladesh. Further, epidemiological data were collected to identify risk factors of the disease that might favor occurrence in the study area.

## Materials and Methods

### Ethical approval and Informed consent

This study did not use any live animal subjects with an invasive method. Therefore, no institutional ethical approval was taken, rather, verbal consent from the animal owners was sought while collecting diarrheic samples/rectal swabs from the dogs.

### Study period and location

The present study was conducted over a period of 3 months (October-December 2019) in the Chattogram metropolitan area (CMA).

### Study design

A cross-sectional study was designed to collect samples and epidemiological data. Individual dogs were considered sampling units. A standard questionnaire was used to collect demographic data, such as local and exotic breeds (Spitz, Lhasa Apso, German shepherd, Labrador Retriever, and Golden Retriever), age (1-6 months and >6 months), sex (male and female), vaccination status (vaccine given or not), and body weight (1-10 kg and >10 kg). Samples were collected from a private veterinary pet clinic in the Chattogram region. Epidemiological data were collected by face-to-face interviews of pet owners and physical examination of the dogs. Clinical signs were observed to identify diseased animals. The case definition for CPV for the present study was the presence of at least four of the following clinical signs: (i) Foul-smelling diarrhea (mucoid to purely hemorrhagic); (ii) vomiting; (iii) anorexia; (iv) depression, lethargy, or weakness; and (v) dehydration [21]. The CMA area was divided into four parts according to ward numbers. One hundred dogs were included in the study. Dogs were categorized as intensive, semi-intensive, and extensive according to ownership, feeding system, housing system, and contact with stray dogs. Intensive management system indicates no contact with stray dogs and keeping the dogs inside the house with owners. Semi-intensive management system indicates that dogs are housed outside the home, allowing frequent contact with stray dogs. Extensive management system indicates that dogs are mainly stray but are given food by owners. No samples were collected from dogs with a history of vaccination in 20 days before sample collection. Vaccine strains might be shed in the post-vaccination period [22-24].

### Sample collection and preservation

Fecal swabs were collected in sterile sampling vials enriched with 1 ml of transport medium containing phosphate-buffered saline (pH 7.2) [25]. Two available canine vaccines were obtained from a vaccine selling agent in Chattogram. Samples and vaccines were transferred to the clinical pathology laboratory of Chattogram Veterinary and Animal Sciences University using an icebox. Samples were preserved at −20°C until further analysis; however, molecular analyses were conducted as soon as possible after sample collection.

### DNA extraction and PCR confirmation of CPV

Fecal samples were thawed at room temperature (25°C) before DNA extraction. Samples were mixed properly using a homogenizer. Forty samples were used for DNA extraction, including 20 suspected cases with clinical signs of disease and 20 randomly selected healthy animals. DNA from fecal samples was extracted using appropriate DNA extraction kits (QIAamp^®^ DNA Stool Mini Kit, Qiagen, Germany) following the manufacturer’s instructions. The boiling method was used to extract DNA from the vaccine [26]. PCR was performed using a primer set coded as CPV(X)-F, CPV(X)-R, CPV-IR (2a), CPV-IF (2b), and CPV-IR (2c), respectively, as reported by a previously published method [13]. In the ARMS-PCR technique, two reaction systems are needed. In the first reaction, the CPV2a variant can be confirmed using CPV(X)-F, CPV(X)-R,CPV-IR (2a), and CPV-IF (2b) primers. Amplified products showed 631 bp+492 bp amplicon for CPV2a and 631 bp+179 bp or 179bp amplicon for CPV2b/CPV2c. A consecutive second reaction is used to differentiate CPV2b and CPV2c using CPV(X)-F, CPV(X)-R, and CPV-IR (2c) primers. An amplicon size of 631 bp+495 bp indicates CPV2c and a single 631 bp amplicon indicates the CPV2b variant [13]. The PCR reaction was set up in 50 mL final volume maintaining standard procedure. The PCR conditions had an initial denaturation step of 94°C for 5 min, followed by 35 cycles of denaturation at 94°C for 30 s, annealing at 51°C for 30 s, extension at 72°C for 45 s, and a final extension step at 72°C for 7 min [13]. Finally, 5 mL of amplicons were taken and stained using 0.05% ethidium bromide (Sigma-Aldrich^®^) followed by visualization of bands on agarose gels (1%).

### Statistical analysis

All data were inserted and coded in a Microsoft Office Excel 2016 spreadsheet and both univariable and multivariable logistic regression analyses were performed using generalized linear mixed models in STATA-IC 13. Statistical analysis was based on the diagnosis of the disease through clinical signs. p≤0.05 was considered statistically significant in both the models. A GIS map of the location of case dogs was created using QGIS 3.12.0.

## Results

### Detection of the CPV variants

**Figure-1:**
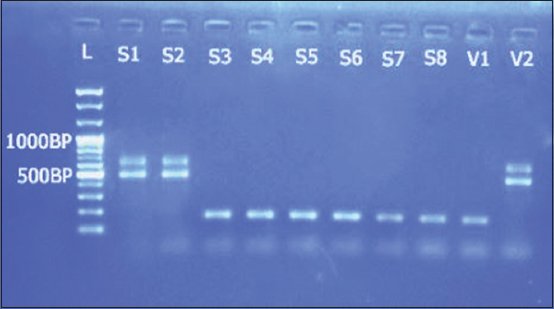
Gel electrophoresis showing amplified product from the samples with CPV(X)-F, CPV(X)-R, CPV-IR (2a), CPV-IF (2b) primers; L=100 bp plus ladder; S1, S2=Field samples (CPV 2a); S3-S8=Field samples (CPV 2b/CPV2c); V1=Vaccine (CPV 2b/CPV2c); V2=Vaccine (CPV 2a).

**Figure-2:**
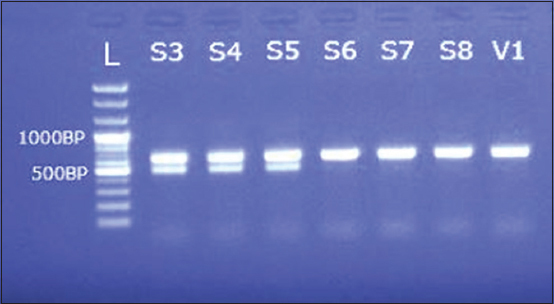
Gel electrophoresis showing amplified product from the samples with CPV(X)-F, CPV(X)-R, CPV-IR(2c) primers L=100 bp plus ladder; S3-S5=Field samples (CPV 2c); S6-S8=Field samples (CPV 2b); V1=Vaccine (CPV 2b).

ARMS-PCR indicated the presence of all the three known variants, CPV2a, CPV2b, and CPV2c, in the collected samples; First time recorded and confirmed by molecular analyses in Bangladesh. The frequencies of occurrence of the variants were 15%, 20%, and 65% for CPV2a, CPV2b, and CPV2c, respectively. In contrast, vaccines showed presence of only the CPV2a and CPV2b variants (Figures-1 and 2).

### Distribution of the variants

**Figure-3:**
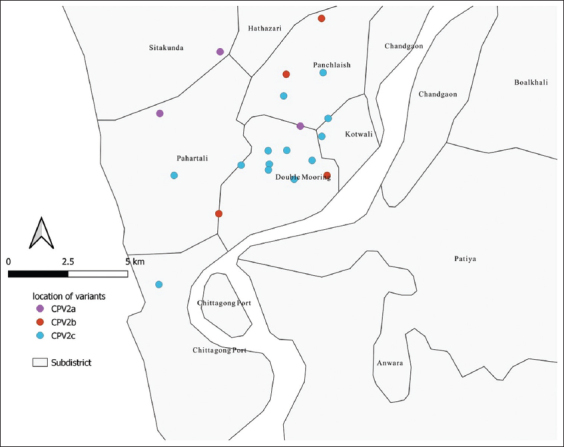
Distribution of the positive samples in the metropolitan area along with the location of different variants [Source: QGIS 3.12.0.].

The GIS map of the location of sampled dogs showed a randomized distribution of cases in the study area (Figure-3). This distribution indicates that the results can be generalized throughout CMA. However, the CPV2c occurrence was clustered at the center of the study area.

### Risk factor analysis

Univariable analysis showed significant relationships among breed, management system, and vaccination status with CPV infection in the study population (Table-1). After accounting for confounding effects of different fixed and random effects in the area using the multivariable linear mixed model, only breed and management system showed significant association with infection (Table-2). Exotic breeds displayed a 3.83-fold higher risk of CPV infection compared with local breeds. Further, dogs reared in semi-intensive and extensive management systems showed 3.64- and 3.79-fold higher probabilities of infection, respectively, than those reared under an intensive management system.

**Table 1:** Prevalence of parvoviral infection and its association with different variables estimated by univariable logistic regression models.

Variable	Category	Observation	Number positive and prevalence (%)	Odds ratio (univariable)	p-value
Breed	Local	54	6 (11.11)	Ref.	0.01
	Exotic	46	14 (30.43)	3.5	
Management system	Intensive	50	6 (12)	Ref.	0.01
	Semi-intensive	22	3 (13.64)	1.15	
	Extensive	28	11 (39.29)	4.74	
Sex	Female	44	11 (25)	Ref.	0.26
	Male	56	9 (16.07)	0.57	
Age	1-6 months	57	12 (21.05)	Ref.	0.76
	>6 months	43	8 (18.60)	0.85	
Weight	1-10 kg	52	12 (23.08)	Ref.	0.42
	>10 kg	48	8 (16.67)	0.66	
Vaccination	Vaccinated	64	8 (12.50)	Ref.	0.01
	Unvaccinated	36	12 (33.33)	3.5	
Month	October	21	4 (19.05)	Ref.	0.87
	November	45	10 (22.22)	1.21	
	December	34	6 (17.65)	0.91	

**Table 2:** Risk factors for parvoviral infection in pet dogs of Chattogram city, October 2019-December 2019 from the final multivariable generalized linear mixed model (logistic regression), taking area as a random effect.

Name of the variables	Level	Estimates	SE**	OR*	CI (95%)	p-value
Intercept		–5.48	1.27			
Breed	Local	0		Ref		0.002
	Others	2.37	1.20	3.83	1.46-6.20	
Management system	Intensive	0		Ref		0.01
	Semi-intensive	1.54	1.41	3.64	0.86-6.42	
	Extensive	1.57	1.13	3.79	1.56-6.01	0.001

* OR=Odds ratio,

** SE=Standard error, CI=Confidence interval

Vaccination showed no significant association with infection in multivariable analysis, but univariable analysis indicated that the disease prevalence was higher (33%) in unvaccinated dogs compared with vaccinated dogs (12%). No significant relationship between gender and CPV was observed, although prevalence was higher in females (Table-1). Other variables, such as age and weight, did not show a significant association with CPV; however, young dogs and dogs with lower weights had a higher prevalence (Table-1).

## Discussion

CPV is present in Bangladesh. CPV2c is the most common variant found in the field and all three variants cause disease in the study area. The most common variant in Asia is CPV2a, whereas CPV2c is the most common variant in Europe, some parts of Asia, and Latin America [4,7,27-30]. A previous study identified CPV2c as the most pathogenic variant, and some confusing reports suggest infection by mixed variants [2]. Further, we observed no significant association between the vaccination status and disease. Therefore, it can be stated that vaccination does not have any effect on the disease occurrence which might be because of using the wrong vaccine variant.

This study spanned only 3 months and was conducted in the southern part of Bangladesh, yet the findings provide a strong foundation for further study. Further, two risk factors were identified with advanced statistical models that might be useful for preventing and controlling infection. Local breeds were less infected with CPV, showing consistency with previous reports [27,31]. Dogs reared under intensive management system were less likely to be infected by CPV, as also reported by Sen *et al*.[19] and Islam *et al*. [3]. We did not observe significant relationships of sex, age, weight, and month of disease occurrence with CPV infection. Some reports indicate that males are at a higher risk of infection [3,17-19,32], and yet another study reports that females are at a higher risk [17]. In some studies, it was observed that juvenile animals were at a higher risk, possibly due to lower levels of maternal antibody and lack of vaccination [2,3]. In the present study, we included only 100 dogs, which limits study power and fails to identify a true association (Type II error) of sex, age, and weight with CPV status. An additional study, including more dogs, is recommended to identify more important risk factors to support effective preventive strategies.

Vaccination is considered to have a significant impact on CPV. Vaccinated animals were probably less infected, as reported in a previous study [32]. However, a considerable number of cases were found where dogs were infected by CPV, despite a history of vaccination [5,27]. As the vaccine variants do not cover all field variants, it may act as a determining factor. Furthermore, different veterinary practitioners use distinct vaccination protocols. Several causes of vaccination failure are known, such as not maintaining cold chain, maternal antibody, worm load, poor nutritional status, lack of protective antibody titers against heterologous CPV antigenic types, and faulty vaccination [5,22,33,34]. Some researchers suggest that the CPV2b vaccine provides cross-protection against CPV2a and CPV2c, but a more intensive study is needed to confirm this possibility [14,33,35-37].

## Conclusion

This study represents a foundation for future CPV studies in Bangladesh and be useful among veterinarians, pet owners, and veterinary disease control authorities.

## Authors’ Contributions

FMYH did all laboratory work and wrote the whole manuscript initially. SA collected the sample, transferred the data in Excel spreadsheet, and helped in writing the article. SC did the data analysis and final revision of the manuscript. FMYH and SA revised the manuscript. All authors read and approved the final manuscript.
